# Simple Cortical and Thalamic Neuron Models for Digital Arithmetic Circuit Implementation

**DOI:** 10.3389/fnins.2016.00181

**Published:** 2016-05-13

**Authors:** Takuya Nanami, Takashi Kohno

**Affiliations:** ^1^Department of Electrical Engineering and Information Systems, Graduate School of Engineering, The University of TokyoTokyo, Japan; ^2^Institute of Industrial Science, The University of TokyoTokyo, Japan

**Keywords:** digital silicon neuronal network, qualitative neuron model, nonlinear dynamics, cerebral cortex, thalamus

## Abstract

Trade-off between reproducibility of neuronal activities and computational efficiency is one of crucial subjects in computational neuroscience and neuromorphic engineering. A wide variety of neuronal models have been studied from different viewpoints. The digital spiking silicon neuron (DSSN) model is a qualitative model that focuses on efficient implementation by digital arithmetic circuits. We expanded the DSSN model and found appropriate parameter sets with which it reproduces the dynamical behaviors of the ionic-conductance models of four classes of cortical and thalamic neurons. We first developed a four-variable model by reducing the number of variables in the ionic-conductance models and elucidated its mathematical structures using bifurcation analysis. Then, expanded DSSN models were constructed that reproduce these mathematical structures and capture the characteristic behavior of each neuron class. We confirmed that statistics of the neuronal spike sequences are similar in the DSSN and the ionic-conductance models. Computational cost of the DSSN model is larger than that of the recent sophisticated Integrate-and-Fire-based models, but smaller than the ionic-conductance models. This model is intended to provide another meeting point for above trade-off that satisfies the demand for large-scale neuronal network simulation with closer-to-biology models.

## 1. Introduction

Silicon neuronal networks have gained remarkable attention in recent years. The silicon neuronal network is composed of dedicated circuit that solves the differential equations of a neuron and synapse model. On account of their parallel and distributed structures, silicon neuronal networks can simulate neuronal activities with low power consumption and in high speed, potentially realizing an extremely large-scale network comparable to that of the human brain in future.

Their analog circuit implementation consumes ultra-low power down to several nano watts per silicon neuron (Brink et al., 2013; Kohno and Aihara, 2014; Mandloi et al., 2014), however, it includes technical hurdles of fabrication mismatch and temperature dependence to construct a large-scale network. On the other hand, digital circuit implementation solves this limitation because it is far less sensitive to these factors, though power consumption tends to be higher than the analog circuit implementations. A digital silicon neuronal network comprising 1 million spiking neurons and 256 million synapses has been implemented on a 5.4-billion transistor chip (Merolla et al., 2014). This Application Specific Integrated Circuit (ASIC) chip calculates asynchronously in real-time and consumes just 63 mW. Merolla et al. adopted the leaky integrate-and-fire (LIF) model, one of the most simple neuronal models. The LIF model describes the dynamics of the neuronal membrane potential, which is perturbed by a stimulus inputs over time and converges slowly to the resting potential. It is computationally efficient and suitable for the large-scale implementation. After refining the synaptic efficacy by a learning process, the ASIC was applied to a multi-object detection and classification.

In silicon neuronal networks, diversified neuronal models are used due to a trade-off between the reproducibility of neuronal activity and computational efficiency. For instance, ionic-conductance models can reproduce a neuronal activity accurately but demands excessive computational resources in large-scale implementations. In contrast, neuronal models that approximate a spiking process by resetting the state variable [integrate-and-fire (I&F) -based models], such as the LIF, exponential I&F (Fourcaud-Trocmé et al., 2003), adaptive exponential I&F (Brette and Gerstner, 2005), and Izhikevich (IZH) models, can be implemented at low computational cost. However, it has reduced reproducibility of complex neuronal activities. For example, these models assume fixed maximum membrane potentials during the spike process, whereas these potentials are nonuniform in the nervous system (Alle and Geiger, 2006). Reduction of computational cost benefits the power consumption, system size, and response speed of silicon neuronal networks regardless of implementation types (ASIC, Field Programmable Gate Array, and massively-parallel CPUs). These factors are particularly important for their application to neuromorphic systems (e.g., for robot control) that are required to occupy a small space and operate with restricted power supply. They are also important to implement with realistic power consumption and system size a large-scale network comparable to the human brain that is composed of about a hundred billion neurons. The DSSN model (Kohno and Aihara, 2007) is a qualitative neuronal model designed for efficient implementation in a digital arithmetic circuit. It is a non-I&F-based model that can realize several neuronal activities including the Class I and II in the Hodgkin's classification (Hodgkin, 1948). Because this model does not abbreviate the calculation of the spiking process, it can reproduce the gradient response in Class II neurons (Wang and Rinzel, 2003). Li et al. (2012) constructed an auto-associative memory with 256 fully connected digital spiking silicon neuron (DSSN) models on an FPGA. They reported that a network of Class II neurons yields higher retrieval performance than that of Class I neurons in the associative memory task. Recently, a Hebbian learning rule was applied to this network (Li et al., 2013). In Osawa and Kohno (2015), it was reported that the Class II mode of the IZH model with a standard parameter setting has discontinuous phase resetting curve (PRC) and the auto-associative memory constructed in the same way does not deliver higher performance than the Class I-mode IZH model. Increasing the value of a parameter can solve this problem, however, it considerably distorts the spiking waveform.

**Figure 1:**
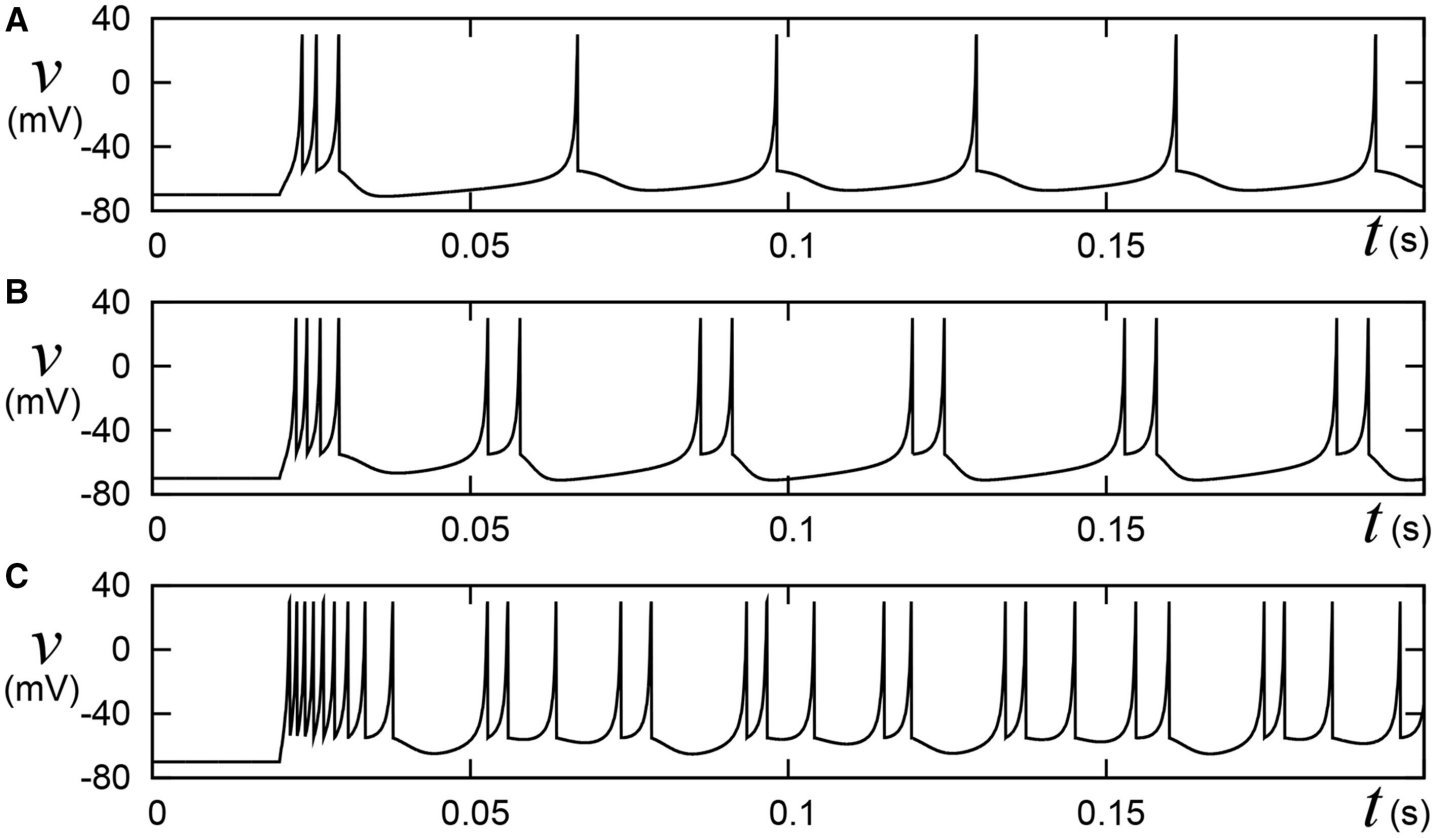
**Behaviors of the Izhikevich model in the IB class**. Stimulus step input *I*_stim_ rise at *t* = 0.02, where **(A)** *I*_stim_ = 10, **(B)** 15, and **(C)** 30, respectively. A numerical integration step *dt* is 0.05 ms.

Pospischil et al. (2008) have found appropriate parameter sets for an ionic-conductance model that replicate experimental data in the well-known four classes of cortical and thalamic neurons; regular spiking (RS), fast spiking (FS), intrinsically bursting (IB), and low-threshold spike (LTS). A most typical cortical neuron class, RS is characterized by spike-frequency adaptation; that is, the spike frequency decreases over time in response to a constant stimulus input. Conversely, FS neurons maintain firing at a constant frequency. IB and LTS are the neuron classes with bursting ability. Neurons in the IB class generate a burst firing immediately at the onset of a stimulus, then continue spiking until its termination. The LTS class also exhibits the bursting. Neurons in the LTS class generate a burst firing just after the termination of a sufficient hyperpolarizing stimulus. Their model equations were constructed by integrating several ionic-conductance models reported in the previous researches and contains up to seven variables. For the RS and FS classes, they applied an auto fitting procedure that minimizes the error function of spike intervals by the simulated annealing method. The IZH model supports these four classes. The Pospischils' ionic-conductance model for the IB class generates a long silent phase following the burst firing (**Figure 11**-left), but the IZH model produced uniform silent phases (Figure 1). Moreover, the response to larger inputs evidently differed from that of Pospischils' model. Because it is not elucidated completely what properties of the neuronal activities are playing the key roles in the information processing in the brain, developing a simple non-I&F model that supports a wide variety of neuronal activities can be significant for both scientific and engineering purposes, even if it consumes computational resources than I&F-based models.

In Nanami and Kohno (2015), we reduced the dimensions of Pospischils models by Keplers method and analyzed their mathematical structures by bifurcation analysis. We also reported parameter sets where the three-variable DSSN model (Kobayashi et al., 2011) produces similar activities to our target classes. While we did not evaluate their similarity, it was clear that the parameter set for the IB class could not reproduce the transient firing patterns from the first bursting phase to the later spiking phase. In this work, we cleared this limitation as follows. Firstly, we repeated the parameter search and found three-variable DSSN model parameter sets for RS, FS, and LTS modes that reproduce corresponding mathematical structures of the reduced Pospischils models and statistical characteristics of spiking patterns. Secondly, for the IB class, we incorporated an additional slow variable and found a parameter set that reproduces corresponding mathematical structures and the statistical characteristics of spiking patterns. The statistical evaluation was performed using Cv and Lv (Shinomoto et al., 2003) that are utilized to characterize the spiking activities of neuronal cells.

The remainder of this paper is organized as follows. Section 2 introduces our neuron model, its basic concepts, and details of its construction. The model is tested and evaluated by simulation in Section 3. Section 4 summarizes the work and suggests ideas for future.

## 2. Methods

In the nervous system, the neuronal cells are classified by their intrinsic spike properties (Llinas, 1988; Connors and Gutnick, 1990; Gupta et al., 2000). The quantitative characteristics of neuronal activity, including the degree of adaptation and duration of the burst firing, differ even among neurons of the same class. To accommodate this fact, Pospischil et al. assembled various experimental data of different nerve cells in each neuron class, and adjusted the parameters of their ionic-conductance models' equations to each dataset. We expanded the DSSN model so that it has a compatible dynamics and behaviors to a typical parameter of the Pospischil's model in each class.

### 2.1. DSSN model

The DSSN model (Kohno and Aihara, 2007) is a qualitative neuron model that can simulate several classes of neuronal activities by Euler's method with fixed point operation. Given appropriate parameter sets, it can reproduce the Class I and II in the Hodgkin's classification as well as Class I^*^ (Fujii and Tsuda 2004) which is defined by a unique mathematical structure named narrow channel. The differential equations of the model is written as follows:

(1) $$\frac{dv}{dt}=\frac{\phi }{\tau }(f(v)-n+I_{0}+I_{\text{stim}}),Ko$$

(2) $$\frac{dn}{dt}=\frac{1}{\tau }(g(v)-n),$$

(3) $$f(v)=\left\{ \begin{array}{l} a_{fn}{\left(v-b_{fn}\right)}^{2}+c_{fn}\;(v<0)\\ a_{fp}{\left(v-b_{fp}\right)}^{2}+c_{fp}\;(v\geq 0), \end{array}\right.$$

(4) $$g(v)=\left\{ \begin{array}{l} a_{gn}{\left(v-b_{gn}\right)}^{2}+c_{gn}\;(v<r_{g})\\ a_{gp}{\left(v-b_{gp}\right)}^{2}+c_{gp}\;(v\geq r_{g}), \end{array}\right.$$

And Kobayashi et al. derived three-variable DSSN model (Kobayashi et al., 2011) that realize autonomous bursting including the square-wave bursting and the elliptic bursting. As explained in the introduction, we reported parameter sets with which this model can produce firing patterns similar to the four classes of cortical and thalamic neuronal activities in Pospischil et al. (2008) but with limited affinity. The equations of this model are given by

(5) $$\frac{dv}{dt}=\frac{\phi }{\tau }(f(v)-n-q+I_{0}+I_{\text{stim}}),$$

(6) $$\frac{dn}{dt}=\frac{1}{\tau }(g(v)-n),$$

(7) $$\frac{dq}{dt}=\frac{\epsilon }{\tau }(h(v)-q),$$

(8) $$h(v)\text{=}\left\{ \begin{array}{l} a_{hn}{\left(v-b_{hn}\right)}^{2}+c_{hn}\;(v<r_{h})\\ a_{hp}{\left(v-b_{hp}\right)}^{2}+c_{hp}\;(v\geq r_{h}), \end{array}\right.$$

where *v* corresponds to the membrane potential, and *n* and *q* are the fast and slow variables, respectively, that abstractly describe the activity of the ion channels. The Parameter *I*_0_ is a bias constant and *I*_stim_ represents the input stimulus. Parameters ϕ, ϵ, and τ control the time constants of the variables. Parameters *r*_*x*_, *a*_*x*_, *b*_*x*_, and *c*_*x*_, where *x* = *fn, fp, gn, gp, hn*, or *hp*, are constants that adjust the nullclines of the variables. All of the variables and constants in this qualitative model are purely abstract with no physical units. Most existing qualitative neuronal models replicate the spiking dynamics by a cubed variable term (FitzHugh, 1961; Nagumo et al., 1962; Hindmarsh and Rose, 1984). Because multiplication consumes significant circuit resources in a digital arithmetic circuit, the DSSN model adopts a piecewise quadratic function so that its numerical integration step includes only one multiplication between variables.

### 2.2. Reduced model

**Figure 2:**
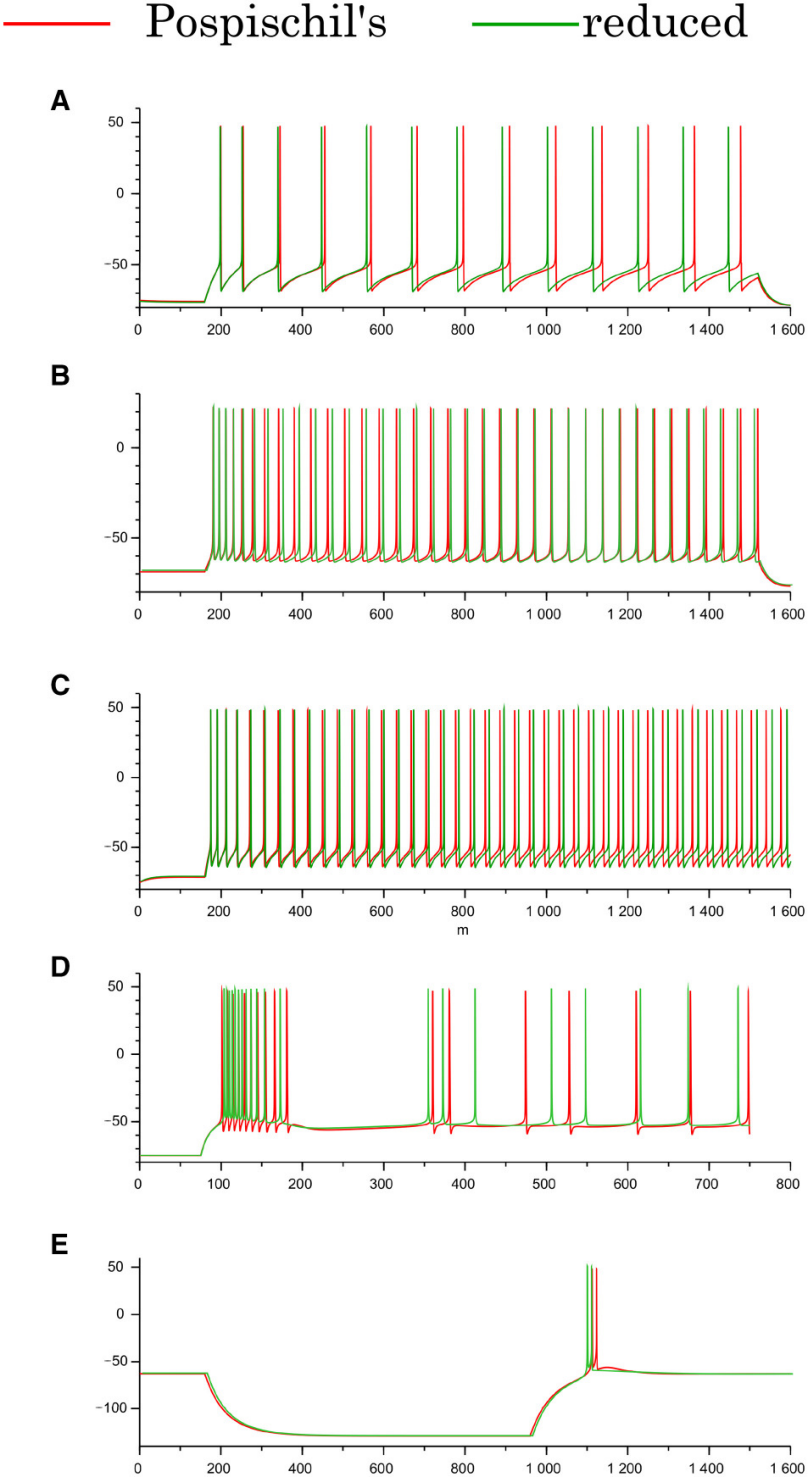
**Waveforms of the Pospischils' and reduced models in the cortical and thalamic neuron classes**. **(A)** The excitatory RS class, **(B)** the inhibitory RS class, **(C)** the FS class, **(D)** the IB class, and **(E)** the LTS class, respectively. An excitatory step stimulus is applied in **(A–D)**. An inhibitory pulse stimulus that starts at 180 ms and ends at 980 ms is applied in **(E)**.

The Pospischils' models contain up to seven variables, which makes an analysis of their mathematical structure difficult. Therefore, by applying Keplers' method (Kepler et al., 1992), we derived three-variable reduced models for the RS, FS, and LTS classes, and a four-variable reduced model for the IB class. Their equations and parameter sets are listed in the Appendix of Supplementary Material. This process reduces the number of variables without critically altering the models' intrinsic structure. The reduced model of the IB class has four variables, because the time-scale differences among the variables prevent the division of the variables into three groups without altering the neuronal activity. Comparing the waveforms of the reduced and Pospischils' models, we confirmed that the dimensional reduction imposed no significant changes on their dynamical properties (Figure 2). In both models, the RS classes have the spike-frequency adaptation (Figures 2A,B). The FS classes have little adaptation. The IB classes have burst firing followed by repetitive single spikes (Figure 2C). The LTS classes generate rebound bursting after removing the long hyperpolarizing stimuli (Figure 2D).

### 2.3. Analysis of mathematical structure

**Figure 3:**
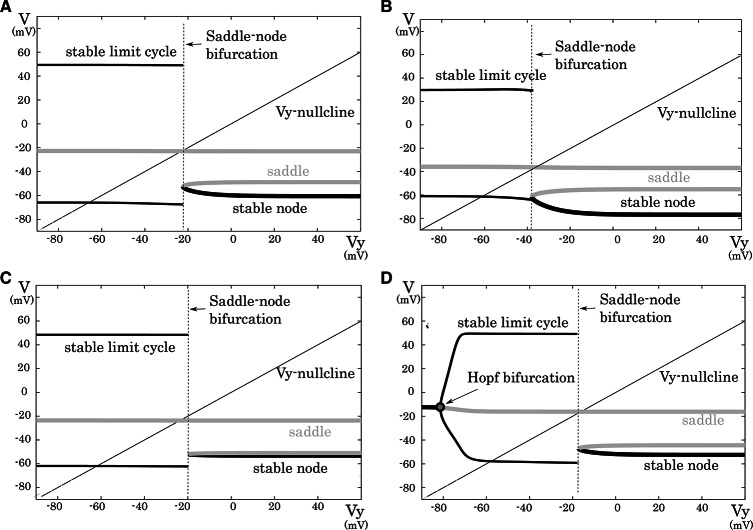
**Mathematical structures of the reduced models**. Projected mathematical structures onto the *V*_*y*_ − *V* plane. A stimulus sufficiently strong for periodic spiking is applied. The saddle-node bifurcation is observed in **(A)** the excitatory RS, **(B)** the inhibitory RS, **(C)** the FS, and **(D)** the LTS classes.

This subsection analyzes mathematical structures of the reduced models derived in the preceding section. The bifurcation diagrams of the reduced models of the excitatory RS, inhibitory RS, FS, and LTS classes are displayed on a *V* – *V*_*y*_ plane with a *V*_*y*_–nullcline (Figure 3). The bifurcation analysis was conducted on the fast subsystem including *V* and *V*_*x*_ while varying the remaining slow variable as a bifurcation parameter. Their mathematical structures are projected onto the *V* − *V*_*y*_ planes. The fast subsystem of these neuron classes undergo a saddle- node on invariant circle bifurcation. Limit cycles which correspond to the periodic spiking are represented by two curves that correspond to their maximum and minimum values of *V*. The *V*_*y*_–nullcline represents the continuous points where $\frac{dV_{y}}{dt}$ is 0. The state point on the stable limit cycle slows down as it approaches to the saddle-node point. Therefore, the slow variable *V*_*y*_ can control the firing frequency, and the variations in the dynamics of *V*_*y*_ induce the different properties of the neuron classes.

**Figure 4:**
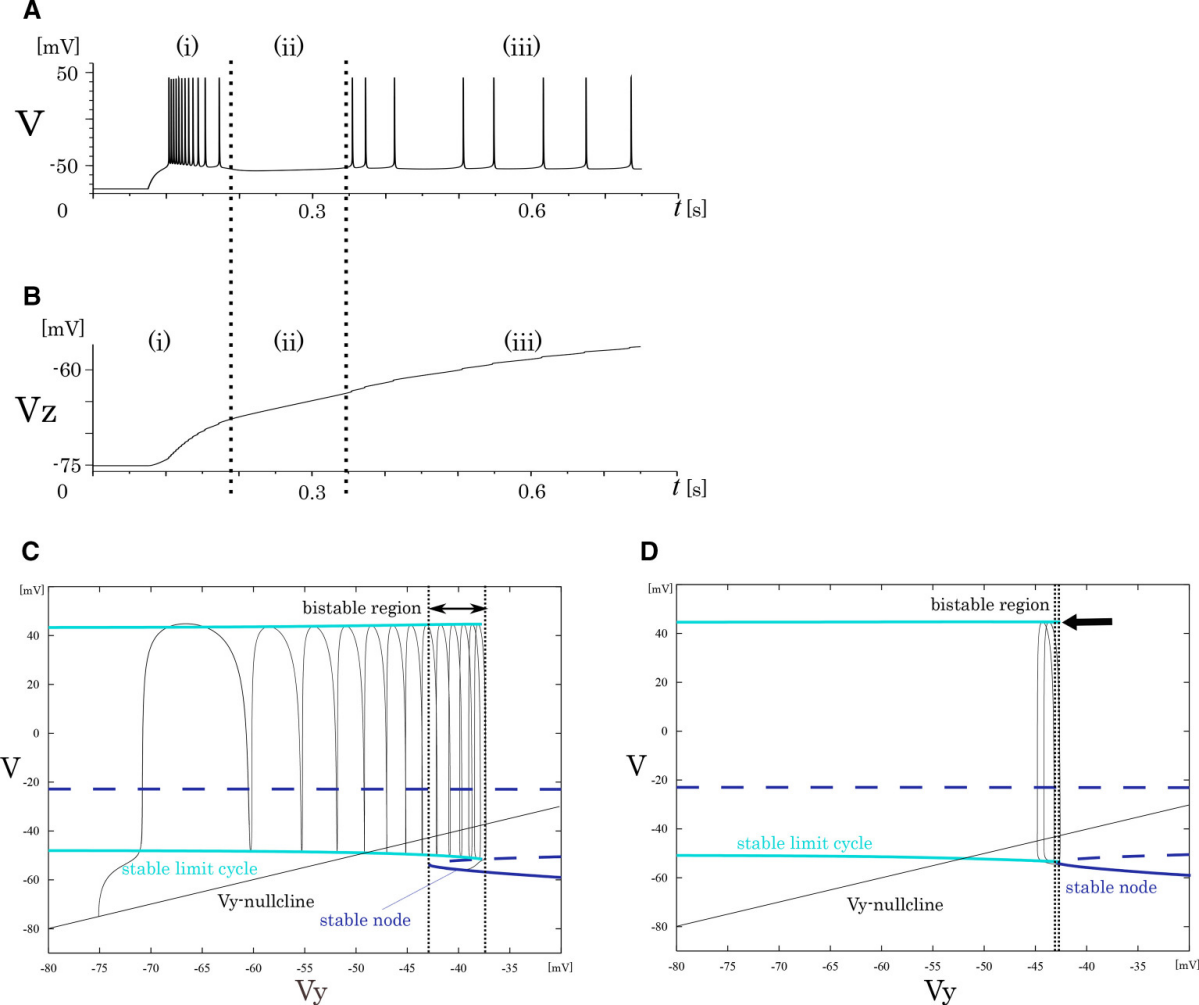
**Mathematical structure of the reduced model in the IB class**. **(A)** Periodic spike waveform of the membrane potential in response to an excitatory step input. **(B)** Transition of the slowest variable *V*_*z*_ that controls the mathematical structure of the fast system. The projected mathematical structure for the **(C)** *V*_*z*_ = –70 mV and **(D)** *V*_*z*_ = −58 mV.

In our analysis of the IB class, we fixed the slowest variable *V*_*z*_ while varying the second slowest variable *V*_*y*_ as the parameter because *V*_*z*_ is sufficiently slower than *V*_*y*_. The transitions in the mathematical structure of the fast subsystem induced by the change in *V*_*z*_ are presented in Figure 4. The fast subsystem exhibits a homoclinic loop bifurcation and the bistable range of *V*_*y*_ depends on the slowest variable *V*_*z*_. Here, the bistable region is where the stable limit cycle overlaps the stable node with respect to the *V*_*y*_-axis. In response to an appropriate step input, the system produces three qualitatively different phases (Figure 4A). They are burst firing, subsequent long silent, and monotonic single spiking phase. This phase transition proceeds as the slowest variable *V*_*z*_ increases (Figure 4B). When *V*_*z*_ is relatively small, a bursting with many spikes is followed by a long silent phase, because the system has a wide bistability region where the state point takes long time to go back to stable limit cycle (Figure 4C). When *V*_*z*_ gets larger, this region is small and the state point can generate only one spike while it is on the limit cycle (Figure 4D).

### 2.4. Parameter tuning and the four-variable DSSN model

**Figure 5:**
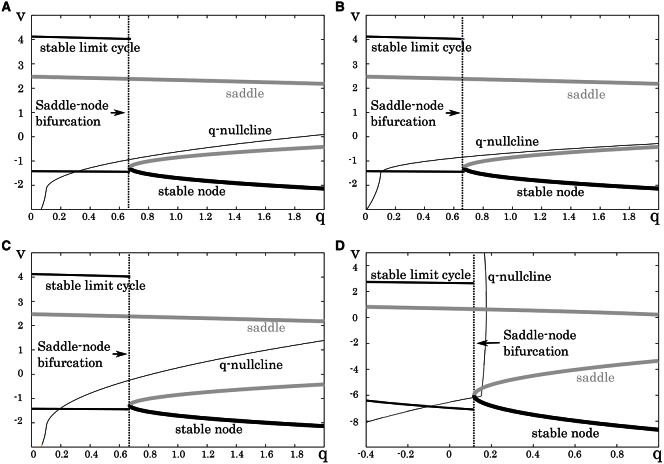
**Mathematical structures of the DSSN models**. Projected mathematical structures onto the *q*-*v* plane. A stimulus sufficiently strong for periodic spiking is applied. A saddle-node on invariant circle bifurcation is seen in **(A)** the excitatory RS, **(B)** inhibitory RS, **(C)** FS, and **(D)** LTS.

To support the RS, FS, and LTS classes by the DSSN model, we found appropriate parameter sets for it that produce the same structures as those in the above reduced models. Firstly, we determined appropriate parameters of the fast subsystem. They were selected so that the system undergoes a saddle-node bifurcation as observed in the reduced models for the RS, FS, and LTS classes (Figure 5). The parameters of the fast subsystem for the LTS mode was tuned to mimic the hyperpolarizing response in the reduced model. Secondly, we tuned the remaining parameters, which control the dynamics of the slow system, to precisely reproduce the distinctive neuronal activities of the corresponding neuron classes. In the RS mode, we placed the *q*-nullcline at relatively low area of the *V*_*y*_ plane so that the system state converges to the limit cycle at near the bifurcation point. It induces spike frequency adaptation because the firing frequency decreases as the slow variable *q* approaches the saddle-node bifurcation point. In the FS mode, the *q*-nullcline was adjusted in the inverse direction to suppress the adaptation. For the LTS mode, the parameters for the fast subsystem were selected to mimic the resting membrane potential in the Pospischils' model which is higher than that in the other modes. Parameters of the slow variable *q* were selected so that it decreases under a hyperpolarizing input. Just after the injection of the hyperpolarizing input has been terminated, because the state point is at far from the saddle-node point, it rapidly moves along the stable limit cycle. This dynamics produces the rebound bursting.

**Figure 6:**
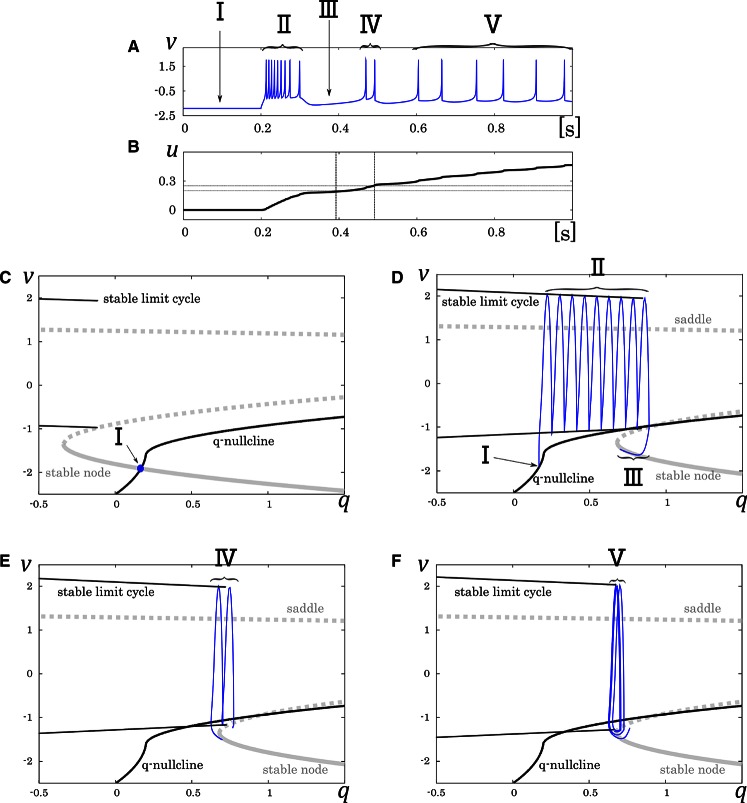
**Mathematical structure of the DSSN model in the IB class**. **(A)** Waveform of the membrane potential in response to a step input that rises at *t* = 0.2 s. **(B)** Transition of the complementary variable u. **(C)** Projected mathematical structure without any input stimulus. Projected mathematical structure under a sustained stimulus where **(D)** *u* < *r*_*u*0_, **(E)** *r*_*u*0_ < *u* < *r*_*u*1_, and **(F)** *r*_*u*1_ < *u*. Each roman number corresponds to a spiking phase.

To support the IB class by the DSSN model, we added a slowest variable in order to vary the bistable area, which was explained in the last part of the previous subsection. The four-variable DSSN model is written as follows:

(9) $$\frac{dv}{dt}=\frac{\phi (u)}{\tau }(f(v)-n-q+I_{0}+I_{\text{stim}})$$

(10) $$\frac{dn}{dt}=\frac{1}{\tau }(g(v)-n)$$

(11) $$\frac{dq}{dt}=\frac{\epsilon }{\tau }(h(v)-q)$$

(12) $$\frac{du}{dt}=\frac{\epsilon_{u}}{\tau }(v-v_{0}-\alpha u)$$

(13) $$\phi (u)=\left\{ \begin{array}{l} \phi_{0}\;(v<r_{u0})\\ \phi_{1}\;(r_{u0}\leq v<r_{u1})\\ \phi_{2}\;(r_{u1}<v) \end{array}\right.$$

where, the new variable *u* dynamically tunes the value of ϕ. Because the equation of *u* does not include multiplication between variables, and ϕ is selected in the list of possible values (ϕ_0_, ϕ_1_, and ϕ_2_), it does not increase the number of multiplications in a numerical integration step. Its mathematical structure is illustrated in Figure 6. With no stimulus, the state point resides at a point which corresponds to the resting state (Figure 6, I). This point is at the intersection of the *q*-nullcline and the stable node. In response to a positive sustained input, the state point jumps into the stable limit cycle and keep firing repetitively (Figure 6, II). The state point drifts rightward because most part of the limit cycle is at above the *q*-nullcline. Therefore, the state point arrives at the right endpoint of the stable limit cycle and falls to the stable node. Subsequently, the state point moves leftward along the stable node because it is at below the *q*-nullcline, which corresponds to the long silent phase (Figure 6, III). Meanwhile, the slowest variable *u* exceeds *r*_*u*0_, and the stable limit cycle shifts leftward. Therefore, the state point goes into the limit cycle again and makes a few spikes along the limit cycle before returning to the stable node (Figure 6, IV). When *u* exceeds *r*_*u*1_, a single spike is generated on the limit cycle before the state point goes back to the stable node (Figure 6, V). The DSSN model qualitatively reproduces the behaviors of the four neuron classes by numerical integration with the Euler's method (*dt* = 0.0001 s).

The DSSN model qualitatively reproduces the behaviors of the four neuron classes by numerical integration with the Euler's method (*dt* = 0.0001 s). For example, the calculation of *v* for IB classes is conducted as follows;

(14) $$v_{\text{next}}=v\_vv+v\_v+v\_n+v\_q+v\_I+v\_c,$$

(15) $$v\_vv_{ij}=v^{2}\;\cdot \;(\Delta t\;\cdot \;a_{fj}\;\cdot \;\phi_{i}/\tau ),$$

(16) $$v\_v_{ij}=v\;\cdot \;(\Delta t\;\cdot \;(-2)\;\cdot \;b_{fj}\;\cdot \;a_{fj}\;\cdot \;\phi_{i}/\tau +1),$$

(17) $$v\_n_{ij}=n\;\cdot \;(-\Delta t\;\cdot \;\phi_{i}/\tau ),$$

(18) $$v\_q_{ij}=q\;\cdot \;(-\Delta t\;\cdot \;\phi_{i}/\tau ),$$

(19) $$v\_I_{ij}=I_{\text{stim}}\;\cdot \;(\Delta t\;\cdot \;\phi_{i}/\tau ),$$

(20) $$v\_c_{ij}=(b_{fj}^{2}\;\cdot \;a_{fj}+c_{fj}+I_{0})\;\cdot \;\Delta t\;\cdot \;\phi_{i}/\tau ,$$

(21) $$v\_x=\left\{ \begin{array}{l} v\_x_{0n}\text{ when }v<r_{u0}\text{ and }v<0\\ v\_x_{1n}\text{ when }r_{u0}\leq v<r_{u1}\text{ and }v<0\\ v\_x_{2n}\text{ when }r_{u1}\leq v\text{ and }v<0\\ v\_x_{0p}\text{ when }v<r_{u0}\text{ and }v\geq 0\\ v\_x_{1p}\text{ when }r_{u0}\leq v<r_{u1}\text{ and }v\geq 0\\ v\_x_{2p}\text{ when }r_{u1}\leq v\text{ and }v\geq 0, \end{array}\right.$$

                                for x=vv, v, n, q, I, and c.

where, *i* denotes 0, 1, or 2 and *j* denotes *n* or *p*. In these equations, we can calculate a product of parameters in advance and store it as a constant value. Parameters are a sum of at most 7 (in average 3.78) positive or negative power-of-two numbers so that the multiplication of a variable and a parameter is realized by combination of bit shift and add operations. Multiplication is only used for the calculation of *v*^2^.

## 3. Results

### 3.1. Comparison of the waveforms

**Figure 7:**
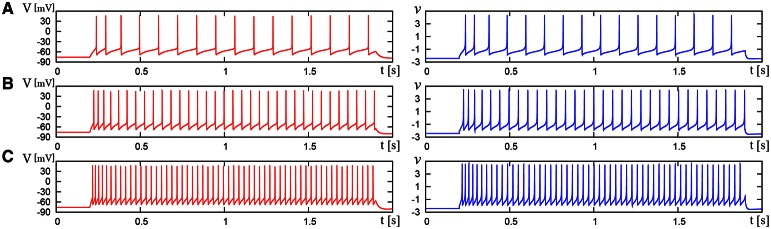
**Waveforms of Pospischil and DSSN models, corresponding to an excitatory neuron in the RS class**. The Pospischils' model (left) generates periodic spikes in response to a step input rising at *t* = 0.2 where *I*_stim_ is **(A)** 1.1, **(B)** 1.5, **(C)** 2.1 nA. The DSSN model (right) generates periodic spikes in response to a step input rising at *t* = 0.2 where *I*_stim_ is **(A)** 2.9221, **(B)** 3.2521, **(C)** 3.7921.

**Figure 8:**
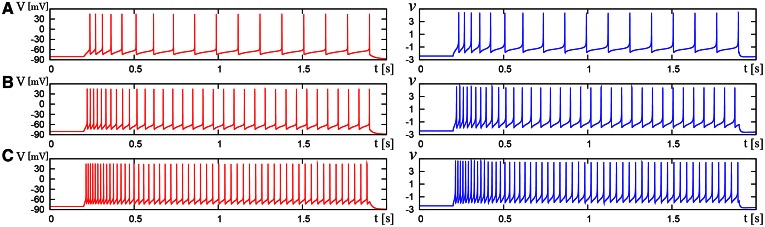
**Waveforms of Pospischil and DSSN models, corresponding to an inhibitory neuron in the RS class**. The Pospischils' model (left) generates periodic spikes in response to a step input rising at *t* = 0.2 where *I*_stim_ is **(A)** 0.6, **(B)** 1.1, **(C)** 1.7 nA. The DSSN model (right) generates periodic spikes in response to a step input rising at *t* = 0.2 where *I*_stim_ is **(A)** 2.97, **(B)** 3.57, **(C)** 4.29.

**Figure 9:**
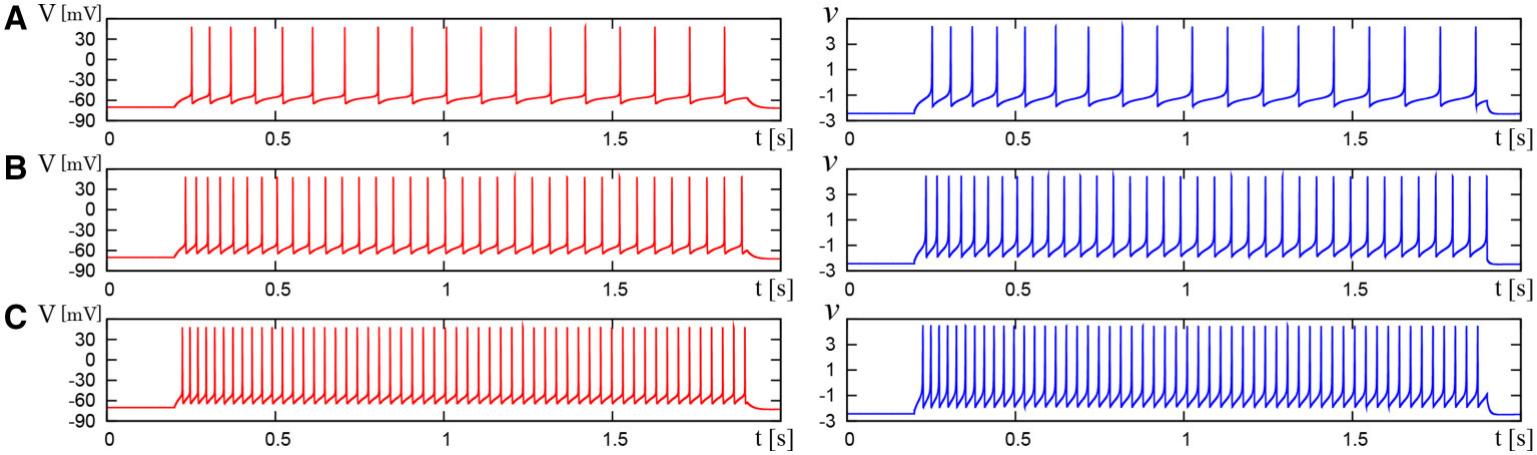
**Waveforms of Pospischil and DSSN models, corresponding to a neuron in the FS class**. The Pospischils' model (left) generates periodic spikes in response to a step input rising at *t* = 0.2 where *I*_stim_ is **(A)** 0.78, **(B)** 0.98, **(C)** 1.22 nA. The DSSN model (right) generates periodic spikes in response to a step input rising at *t* = 0.2 where *I*_stim_ is **(A)** 2.7, **(B)** 2.95, **(C)** 3.25.

**Figure 10:**
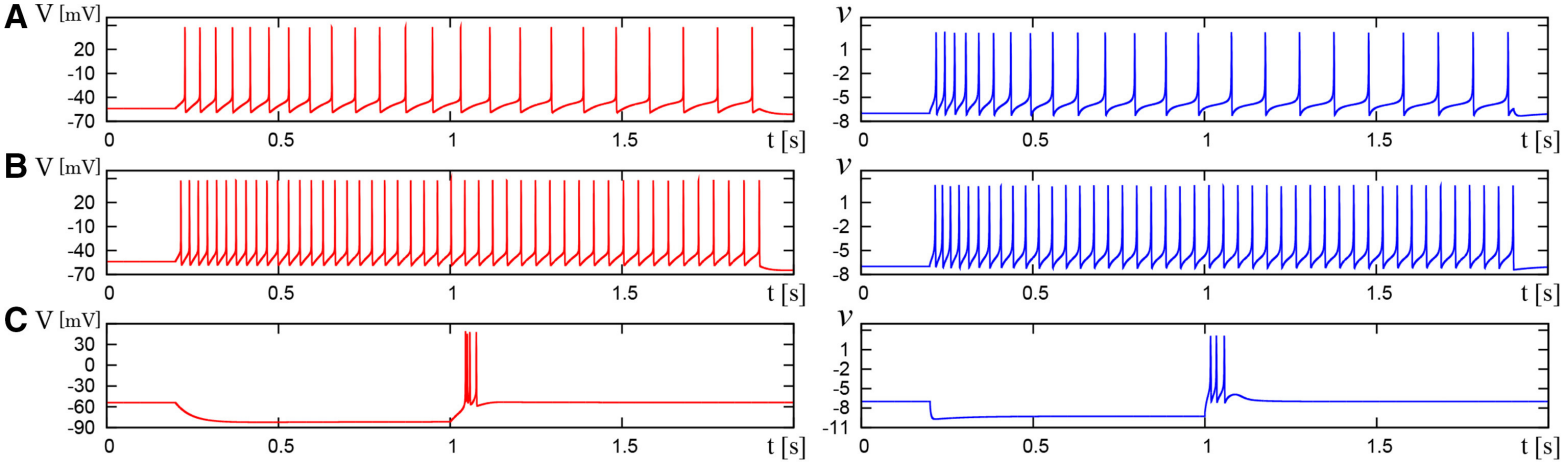
**Waveforms of Pospischil and DSSN models, corresponding to a neuron in the LTS class**. The Pospischils' model (left) generates periodic spikes in response to a step input rising at *t* = 0.2 where *I*_stim_ is **(A)** 0.34, **(B)** 0.79, **(C)** –0.6 nA. The DSSN model (right) generates periodic spikes in response to a step input rising at *t* = 0.2 where *I*_stim_ is **(A)** 0.278, **(B)** 0.308, **(C)** –1.7. **(A,B)** Pospischils' model (left) and DSSN model (right) generate periodic spike in response to a depolarizing step input rising at *t* = 0.2. **(C)** A hyperpolarizing step input is applied from *t* = 0.2 to *t* = 1.0.

**Figure 11:**
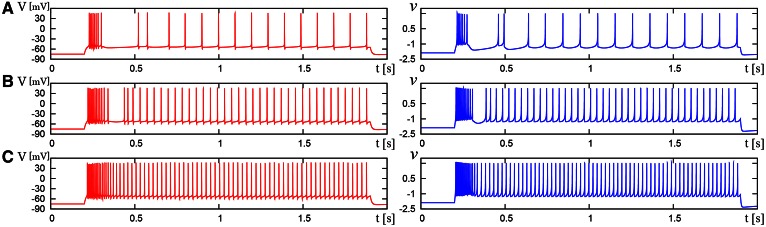
**Waveforms of Pospischils' model and DSSN model which correspond to a neuron in the IB class**. The Pospischils' model (left) generates periodic spikes in response to a step input rising at *t* = 0.2 where *I*_stim_ is **(A)** 2.38, **(B)** 2.6645, **(C)** 3.0495 nA. The DSSN' model (right) generates periodic spikes in response to a step input rising at *t* = 0.2 where *I*_stim_ is **(A)** 0.89, **(B)** 1.42, **(C)** 1.81.

We compared the waveforms generated by the DSSN and Pospischils' ionic-conductance models for several magnitudes of step inputs. The DSSN model accurately reproduce Pospischils' model in each neuron class (Figures 7–**11**). In the excitatory RS mode, both models exhibit the spike-frequency adaptation, and their firing frequencies increase as input stimuli become large (Figure 7). Figure 8 shows the inhibitory RS mode that has more stronger spike-frequency adaptation than the excitatory RS mode. Both models in the FS mode fire with almost constant frequency, which increases as the stimulus is strengthened. In the LTS mode (Figure 10), both models generate periodic spiking with spike-frequency adaptation in response to positive step stimulus and a rebound bursting at the end of a negative stimulus. Both models in the IB mode exhibit repetitive spiking following a burst firing in response to a weak positive input (Figure 11). The frequency of the repetitive spiking gets higher in response to a stronger positive stimulus.

### 3.2. *C*_*V*_ − *L*_*V*_

**Figure 12:**
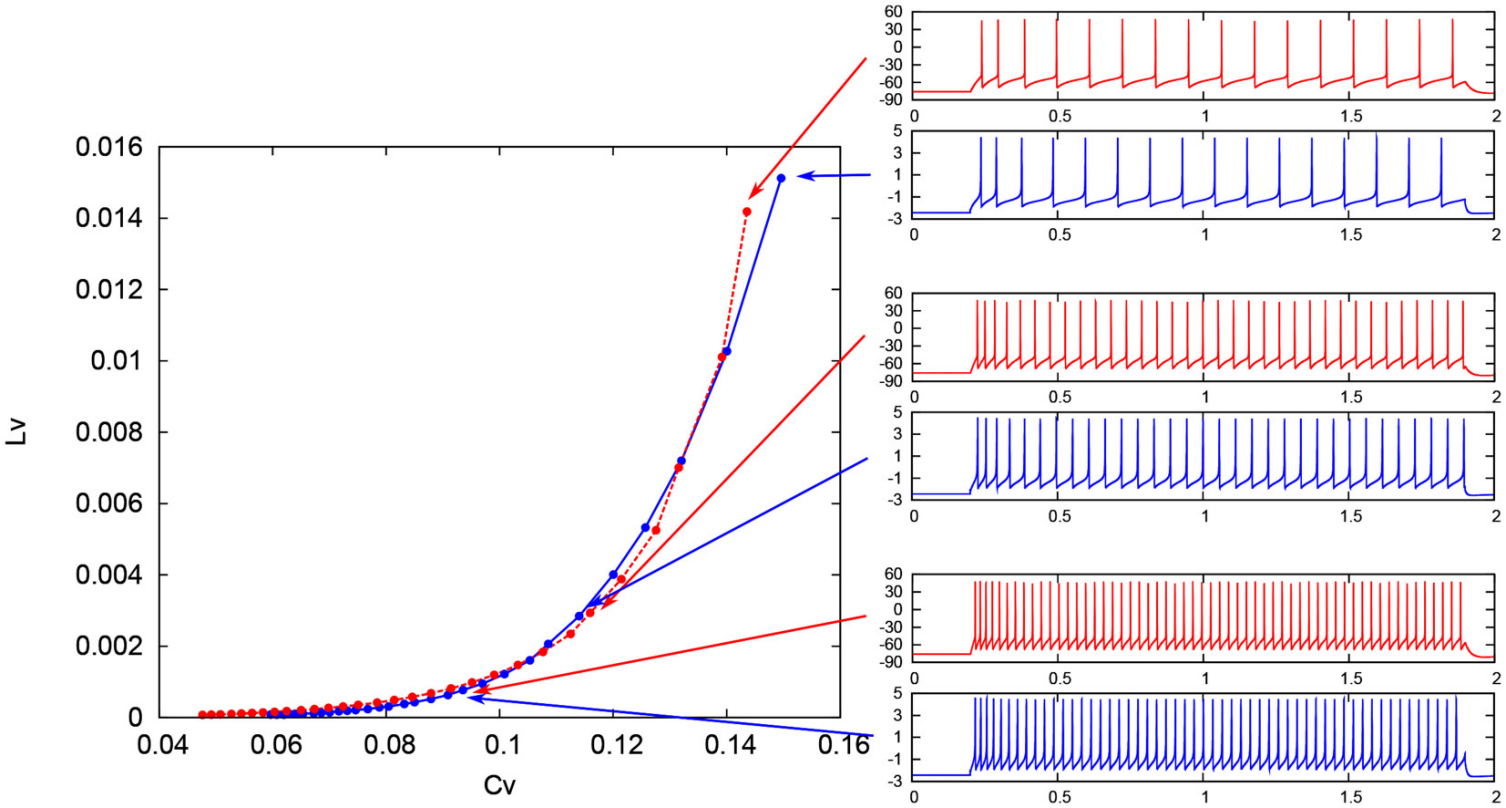
**Spike sequences and their representation in the *C*_*V*_ − *L*_*V*_ plane**. The *L*_*V*_ vs. *C*_*V*_ for spike sequences generated by the Pospischil (red) and the DSSN (blue) models in the excitatory RS mode in response to various magnitudes of step input.

**Figure 13:**
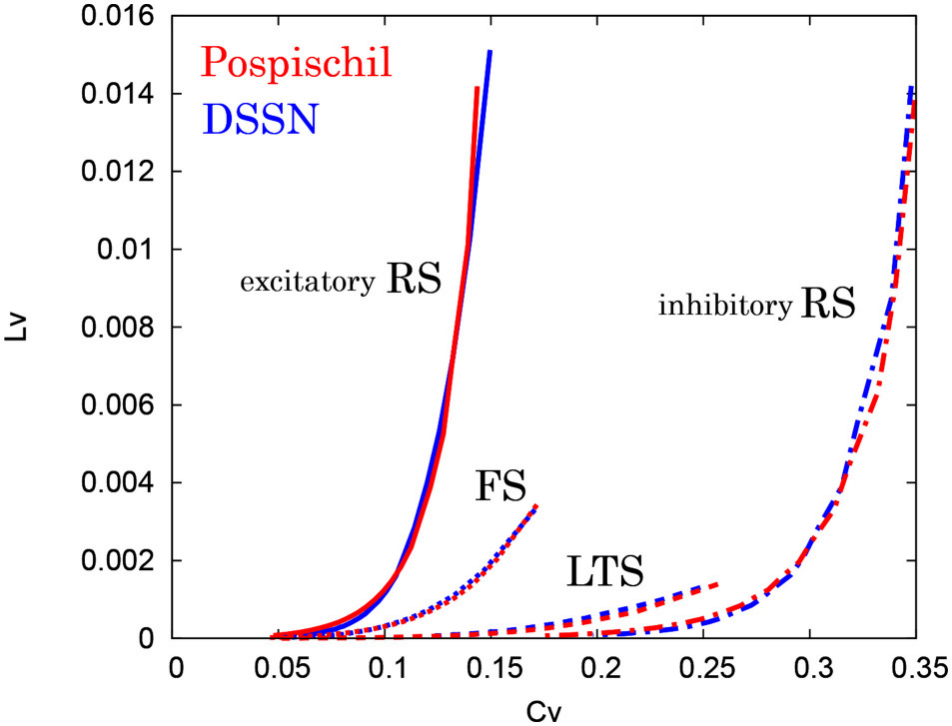
***C*_*V*_ − *L*_*V*_ characteristics in the RS, FS, and LTS classes**. The *C*_*V*_ − *L*_*V*_ characteristics of the Pospischil (red) and the DSSN (blue) models in the excitatory and inhibitory RS, FS, and LTS classes.

**Figure 14:**
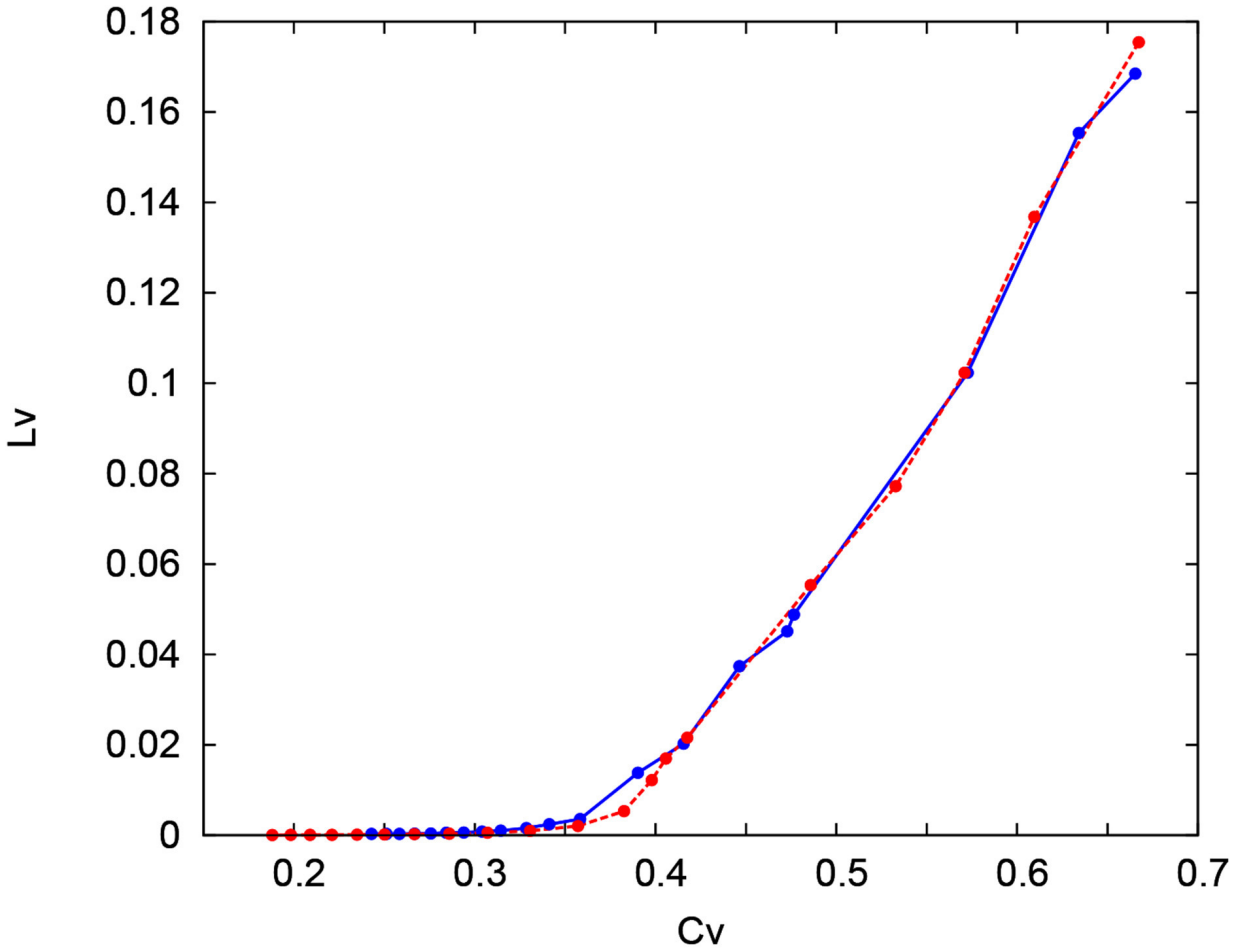
***C*_*V*_ − *L*_*V*_ characteristics in the IB classes**. The *C*_*V*_ − *L*_*V*_ characteristics of the Pospischil (red) and DSSN (blue) model.

To evaluate quantitatively our DSSN models, we adopted the coefficient of variation (*C*_*V*_) and local variation (*L*_*V*_) (Shinomoto et al., 2003), because the spike frequency cannot capture the spiking characteristics that changes over time. These statistics were developed by Shinomoto et al. to classify the spike sequences in the cortical areas of awake monkeys (Shinomoto et al., 2003), which have been used at various situations (Koyama and Shinomoto, 2005; Miura et al., 2006; Shinomoto et al., 2009). They are defined as follows:

(22) $$C_{V}=\sqrt{\frac{1}{n-1}\sum_{i=1}^{n}{\left(T_{i}-\bar{T}\right)}^{2}}/\bar{T},$$

(23) $$L_{V}=\frac{1}{n-1}\sum_{i=1}^{n-1}\frac{3{\left(T_{i}-T_{i+1}\right)}^{2}}{{\left(T_{i}+T_{i+1}\right)}^{2}},$$

where *T*_*i*_ denotes the *i*th interspike interval, $\bar{T}$ is the average of *T*_*i*_, and *n* is the number of spikes in the sequence. The coefficient 3 in Equation (21) is chosen so that the expectation value of *L*_*V*_ in the Poisson spike sequence equals one. The *C*_*V*_ is the standard deviation divided by the mean spike interval, and *L*_*V*_ becomes large when the duration of the spike intervals locally fluctuates. Both of *C*_*V*_ and *L*_*V*_ are zero for a regular spike sequence with constant *T*_*i*_. We measured the *C*_*V*_ and *L*_*V*_ of the spike sequences generated by Pospischils' and our DSSN models while varying the strength of the step input. As shown in the Figure 12, the results are compared by their plot on the *C*_*V*_ − *L*_*V*_ plane. The *C*_*V*_ − *L*_*V*_ characteristics of the Pospischils' and the DSSN models in each neuron class are plotted in Figures 13, 14. We confirmed that both neuron models have similar statistical properties in each neuron class.

## 4. Conclusion

In this paper, we expanded the DSSN model so that it can support the RS, FS, LTS, and IB classes, according to the following steps. Firstly, we reduced the number of variables of the Pospischils' ionic-conductance model utilizing the Keplers' method. The reduced models contained three variables for the RS, FS, and LTS classes and four variables for the IB class. We then elucidated their mathematical structures, including their structures on the phase portraits. The FS class is generically considered to exhibit a Hopf bifurcation; however, it yielded a saddle-node bifurcation in the Pospischils' model. Secondly, we determined the appropriate parameter sets that produce the same mathematical structures in the DSSN model as those in the reduced models. To support the IB class, the four-variable DSSN was developed by adding a slowest variable. It dynamically tunes the structure of the fast system. If ϕ_0_, ϕ_1_, and ϕ_2_ are equal to ϕ, the four-variable DSSN model is compatible with the three-variable one. Finally, it was confirmed that the DSSN models behave very similarly to the Pospischils' models in response to several magnitudes of step input. The similarity was quantitatively confirmed by measuring the *C*_*V*_ and *L*_*V*_ statistics of the spike sequences, and confirming that both neuron models have the same statistical properties in each neuron class. The model is solved by Euler's method (Δ*t* = 0.0001[s]), and is realized by only one multiplier that consumes significant digital circuit resources.

The DSSN model will consume more circuit resources than the IZH model because it has more variables, while the number of multiplication per a numerical integration step is the same. But it captures more aspects of the neuronal activity because it is not an I& F-based model. As is apparent from the equations, the implementation cost of the DSSN model is far lower than the ionic-conductance models. Circuit implementation of this model will be reported in our future publication. In software simulation, the computational cost of the DSSN mode is much lower than that of the ionic-conductance models, and comparable with that of the IZH per a numerical integration step. We conducted simulation of two million spikes, the DSSN and IZH models in the RS mode consumed 18.51 and 2.76 s, respectively. The DSSN model required over five times longer calculation, because the step of the DSSN model (0.0001[s]) is five times smaller than that of the IZH model (0.0005[s]). If the fixed point operation is used, it will be simulated faster. The DSSN model may be also useful for computer simulation in which more realistic model than I&F-based models are required.

By the expansion in this work, the DSSN model supports the RS, FS, LTS, and IB classes as well as the Class I and II in the Hodgkin's classification, Class I^*^, square-wave bursting, elliptic bursting. We expect it can be a basis for silicon- and software- based spiking neuronal networks that capture the dynamics in the nervous system approximately, which can contribute to the neuroscience from the viewpoint of analysis by construction and the neuromorphic engineering. In this work, we tuned the parameters manually. We will work on an auto-fitting method for the DSSN model with an error function based on the mathematical structure.

## Author contributions

TN performed the study and TK supervised it.

### Conflict of interest statement

The authors declare that the research was conducted in the absence of any commercial or financial relationships that could be construed as a potential conflict of interest.
