# Supplementary material for: Simple Cortical and Thalamic Neuron Models for Digital Arithmetic Circuit Implementation
Source: Front Neurosci. 2016 May 13;10:181. doi: 10.3389/fnins.2016.00181 (PMC4865656; doi:10.3389/fnins.2016.00181)
Supplement: Supplementary file 1 [file DataSheet1.pdf]

APPENDIX

The differential equations of the reduced model are as follows:

$$C_{\mathrm{m}}\frac{dV}{dt} = (-g_{\mathrm{leak}}(V - E_{\mathrm{leak}}) - I_{\mathrm{Na}}(V, G_m(V), G_h(V_x)) - I_{\mathrm{Kd}}(V, G_n(V_x)) - I_{\mathrm{M}}(V, p_\infty(V_y))$$
$$-I_{\mathrm{T}}(V, u_\infty(V_y)) - I_{\mathrm{L}}(V, G_q(V_x), G_r(V_z)) + I_{\mathrm{stim}}),$$

$$\frac{dV_x}{dt} = H_h(V)f_h(V, V_x) + H_n(V)f_n(V, V_x) + H_q(V)f_q(V, V_x),$$
$$\frac{dV_y}{dt} = H_p(V)f_p(V, V_y) + H_u(V)f_u(V, V_y),$$
$$\frac{dV_z}{dt} = H_r(V)f_r(V, V_z),$$

where, V denotes a membrane potential. V_x, V_y, and V_z represent the fast, slow, and slowest state variables, respectively. G is a function that converts the state variable to the gating variable. Its subscripts, m, h, n, p, q, r, and u correspond to those gating variables in the Pospischil's ionic-conductance model.

C_{m} is the capacitance of the membrane, g_{leak} and E_{leak} are conductance and reversal potential of the leak current, and I_{stim} is an input stimulus. The functions f_i that control the differential equations of the state variables are

$$f_i(V, V_j) = (\alpha_i(V) + \beta_i(V))\Big(G_i(V) - G_i(V_j)\Big)\frac{d\,G_i(V_j)}{dV_j},$$

$$f_p(V, V_j) = (p_\infty(V) - p_\infty(V_j))/\tau_p(V)\frac{d\,p_\infty(V_j)}{dV_j},$$

$$f_u(V, V_j) = (u_\infty(V) - u_\infty(V_j))/\tau_u(V)\frac{d\,u_\infty(V_j)}{dV_j},$$

$$G_i(V) = \frac{\alpha_i(V)}{\alpha_i(V) + \beta_i(V)},$$

where i is h, n, q, or r and j is x, y, or z. Coefficients $H_x(V)$ ($x = h, n, p, q, r,$ or u) for each neuron class are listed in Table 6-10. The functions for the voltage-dependent ionic currents are

$$I_{\mathrm{Na}}(V, x_1, x_2) = \bar{g}_{\mathrm{Na}} {x_1}^3 x_2 (V - E_{\mathrm{Na}}),$$

$$I_{\mathrm{Kd}}(V, x_1) = \bar{g}_{\mathrm{Kd}} {x_1}^4 (V - E_{\mathrm{K}}),$$

$$I_{\mathrm{M}}(V, x_1) = \bar{g}_{\mathrm{M}} x_1 (V - E_{\mathrm{K}}),$$

$$I_{\mathrm{L}}(V, x_1, x_2) = \bar{g}_{\mathrm{L}} {x_1}^2 x_2 (V - E_{\mathrm{Ca}}),$$

$$I_{\mathrm{T}}(V, x_1) = \bar{g}_{\mathrm{T}} s_\infty^2 x_1 (V - E_{\mathrm{Ca}}),$$

where

$$\alpha_m(V) = \frac{-0.32(V - V_{\mathrm{T}} - 13)}{\exp[-(V - V_{\mathrm{T}} - 13)/4] - 1},$$

$$\beta_m(V) = \frac{0.28(V - V_{\mathrm{T}} - 40)}{\exp[(V - V_{\mathrm{T}} - 40)/5] - 1},$$

$$\alpha_h(V) = 0.128 \exp[-(V - V_{\mathrm{T}} - 17)/18],$$

$$\beta_h(V) = \frac{4}{1 + \exp[-(V - V_{\mathrm{T}} - 40)/5]},$$

$$\alpha_n(V) = \frac{-0.032(V - V_T - 15)}{0.5\exp[-(V - V_T - 15)/5] - 1},$$

$$\beta_n(V) = 0.5\exp[-(V - V_T - 10)/40],$$

$$p_\infty(V) = \frac{1}{1 + \exp[-(V + 35)/10]},$$

$$\tau_p(V) = \frac{\tau_{max}}{3.3\exp[(V + 35)/20] + \exp[-(V + 35)/20]},$$

$$\alpha_q(V) = \frac{0.055(-27 - V)}{\exp[(-27 - V)/3.8] - 1},$$

$$\beta_q(V) = 0.94\exp[(-75 - V)/17],$$

$$\alpha_r(V) = 0.000457\exp[(-13 - V)/50],$$

$$\beta_r(V) = \frac{0.0065}{\exp[(-15 - V)/28] + 1},$$

$$s_\infty(V) = \frac{1}{1 + \exp[-(V + V_a + 57)/6.2]},$$

$$u_\infty(V) = \frac{1}{1 + \exp[(V + V_a + 81)/4]},$$

$$\tau_u(V) = \frac{30.8 + (211.4 + \exp[(V + V_a + 113.2)/5])}{3.7(1 + \exp[(V + V_a + 84)/3.2])}.$$
