# Supplementary material for: Simple Cortical and Thalamic Neuron Models for Digital Arithmetic Circuit Implementation
Source: Front Neurosci. 2016 May 13;10:181. doi: 10.3389/fnins.2016.00181 (PMC4865656; doi:10.3389/fnins.2016.00181)
Supplement: Supplementary file 2 [file Table1.pdf]

Supplementary Material: Simple cortical and thalamic neuron models for digital arithmetic circuit implementation

Takuya Nanami [1,*], **Takashi Kohno** [2]
*Correspondence:
Author Name:
nanami@sat.t.u-tokyo.ac.jp

1 SUPPLEMENTARY TABLES

Table 1. Parameter set for the excitable RS mode of the DSSN model

Par.	Value	Par.	Value
a_{fn}	4.0045619011	a_{fp}	-0.25
b_{fn}	-0.3000113666	b_{fp}	4.8056564331
c_{fn}	0.2891974151	c_{fp}	6.4232187271
a_{gn}	2.1983966827	a_{gp}	15.9919834137
b_{gn}	0.5	b_{gp}	2.6564538479
c_{gn}	-9.9944877625	c_{gp}	1.8500213623
a_{hn}	-0.0317164175	a_{hp}	0.3619402945
b_{hn}	-1.9117646217	b_{hp}	-2.1958761215
c_{hn}	0.1009931862	c_{hp}	0.0961881876
r_g	3	r_h	-2.1700000763
ϕ	1.0981963873	ϵ	0.0167835671
τ	0.0016416833	I_0	-9.5

Table 2. Parameter set for the inhibitory RS mode of the DSSN model

Par.	Value	Par.	Value
a_{fn}	4.0074076653	a_{fp}	-0.25
b_{fn}	-0.3000230789	b_{fp}	4.8092589378
c_{fn}	0.2818711996	c_{fp}	6.4248361588
a_{gn}	2.1991870403	a_{gp}	15.9959344864
b_{gn}	0.5	b_{gp}	2.6564166546
c_{gn}	-9.9969511032	c_{gp}	1.8555984497
a_{hn}	-0.0312500037	a_{hp}	1.2544642687
b_{hn}	-1.25	b_{hp}	-1.5088968277
c_{hn}	0.1001674235	c_{hp}	0.0925347805
r_g	3	r_h	-1.5
ϕ	1.0975610018	ϵ	0.0035569104
τ	0.0016650406	I_0	-9.5

Table 3. Parameter set for the FS mode of the DSSN model

Par.	Value	Par.	Value
a_{fn}	4.0045619011	a_{fp}	-0.25
b_{fn}	-0.2999544442	b_{fp}	4.8047447205
c_{fn}	0.2893342078	c_{fp}	6.4210281372
a_{gn}	2.1963927746	a_{gp}	15.9919834137
b_{gn}	0.5	b_{gp}	2.6563909054
c_{gn}	-9.9919834137	c_{gp}	1.8553695679
a_{hn}	-0.0309734493	a_{hp}	0.1438053101
b_{hn}	-1.9642858505	b_{hp}	-2.2346153259
c_{hn}	0.1040218174	c_{hp}	0.0960667133
r_g	3	r_h	-2.17
ϕ	1.0981963873	ϵ	0.0070766532
τ	0.0016416833	I_0	-9.5

Table 4. Parameter set for the LTS mode of the DSSN model

Par.	Value	Par.	Value
a_{fn}	0.2500000298	a_{fp}	-1.0002056360
b_{fn}	-4.0008220673	b_{fp}	1
c_{fn}	0.9984374046	c_{fp}	6.0002875328
a_{gn}	0.1239570901	a_{gp}	0.4982121587
b_{gn}	-2.0096154213	b_{gp}	-2.7583732605
c_{gn}	-4.0000114441	c_{gp}	-3.9146656990
a_{hn}	0.1222209111	a_{hp}	-0.0005070860
b_{hn}	-9.4002103806	b_{hp}	0.5974025726
c_{hn}	-0.9002342224	c_{hp}	0.2249979228
r_g	-3	r_h	-6.4000000954
ϕ	2.8986887932	ϵ	0.0110465623
τ	0.0009764004	I_0	-4.0999999046

Table 5. Parameter set for the IB mode of the DSSN model

Par.	Value	Par.	Value
a_{fn}	4.01612854	a_{fp}	-0.5020160675
b_{fn}	-0.2999498546	b_{fp}	2.3995988369
c_{fn}	0.2711298466	c_{fp}	3.523106575
a_{gn}	2.3982989788	a_{gp}	19.9957485199
b_{gn}	0.4001182318	b_{gp}	0.752038002
c_{gn}	-9.9984130859	c_{gp}	-9.6617603302
a_{hn}	-0.1875	a_{hp}	1.5833332539
b_{hn}	-1.4999998808	b_{hp}	-1.6118421555
c_{hn}	0.1927082688	c_{hp}	0.1781110764
r_g	0.8000000119	r_h	-1.6000000238
ϵ	0.0021261517	ϵ_u	0.0008211879
τ	0.0005805811	I_0	-7.6999998093
r_{u0}	0.2	r_{u1}	0.23
ϕ_0	0.351523757	ϕ_1	0.3685329854
ϕ_2	0.3883770704	v_0	-1.9133889675
α	1.0477325916		

Table 6. Parameter set for the excitable RS mode of the reduced model

Par.	Value	Par.	Value
$H_h(V)$	$\frac{1}{1+\exp[0.5(V+20)]}$	$H_n(V)$	$1-\frac{1}{1+\exp[0.5(V+20)]}$
$H_q(V)$	0	$H_p(V)$	1
$H_u(V)$	0	$H_r(V)$	0
g_{leak}	0.0205	$\bar{g}_{\text{Na}}$	56
$\bar{g}_{\text{Kd}}$	6	V_{T}	-56.2
$\bar{g}_{\text{M}}$	0.075	τ_{max}	608
$\bar{g}_{\text{L}}$	0	$\bar{g}_{\text{T}}$	0
E_{leak}	-75	C_m	1
E_{Na}	50	E_{K}	-90
E_{Ca}	120	V_a	2

Table 7. Parameter set for the inhibitory RS mode of the reduced model

Par.	Value	Par.	Value
$H_h(V)$	$\frac{1}{1+\exp[0.5(V+20)]}$	$H_n(V)$	$1-\frac{1}{1+\exp[0.5(V+20)]}$
$H_q(V)$	0	$H_p(V)$	1
$H_u(V)$	0	$H_r(V)$	0
g_{leak}	0.0133	$\bar{g}_{\text{Na}}$	10
$\bar{g}_{\text{Kd}}$	2.1	V_{T}	-67.9
$\bar{g}_{\text{M}}$	0.098	τ_{max}	934
$\bar{g}_{\text{L}}$	0	$\bar{g}_{\text{T}}$	0
E_{leak}	-56.2	C_m	1
E_{Na}	50	E_{K}	-90
E_{Ca}	120	V_a	2

Table 8. Parameter set for the FS mode of the reduced model

Par.	Value	Par.	Value
$H_h(V)$	0.5	$H_n(V)$	0.5
$H_q(V)$	0	$H_p(V)$	1
$H_u(V)$	0	$H_r(V)$	0
g_{leak}	0.038	$\bar{g}_{\text{Na}}$	58
$\bar{g}_{\text{Kd}}$	3.9	V_{T}	-57.9
$\bar{g}_{\text{M}}$	0.0787	τ_{max}	502
$\bar{g}_{\text{L}}$	0	$\bar{g}_{\text{T}}$	0
E_{leak}	-70.4	C_m	1
E_{Na}	50	E_{K}	-90
E_{Ca}	120	V_a	2

Table 9. Parameter set for the LTS mode of the reduced model

Par.	Value	Par.	Value
$H_h(V)$	0.5	$H_n(V)$	0.5
$H_q(V)$	0	$H_p(V)$	$\frac{1}{1+\exp[V+70]}$
$H_u(V)$	$1-\frac{1}{1+\exp[V+70]}$	$H_r(V)$	0
g_{leak}	0.019	$\bar{g}_{\text{Na}}$	50
$\bar{g}_{\text{Kd}}$	4	V_{T}	-50
$\bar{g}_{\text{M}}$	0.028	τ_{max}	4000
$\bar{g}_{\text{L}}$	0	$\bar{g}_{\text{T}}$	0.4
E_{leak}	-50	C_m	1
E_{Na}	50	E_{K}	-90
E_{Ca}	120	V_a	-7

Table 10. Parameter set for the IB mode of the reduced model

Par.	Value	Par.	Value
$H_h(V)$	$\frac{1.9125}{1+\exp[-0.25(V+45))}$	$H_n(V)$	$1 - \frac{1.9125}{1+\exp[-0.25(V+45))} - 2.04exp\left(\frac{-(V+52)^2}{18}\right)$
$H_q(V)$	$2.04\,exp\left(\frac{-(V+52)^2}{18}\right)$	$H_p(V)$	1
$H_u(V)$	0	$H_r(V)$	1
g_{leak}	0.1	$\bar{g}_{\text{Na}}$	50
$\bar{g}_{\text{Kd}}$	4.2	V_{T}	-58
$\bar{g}_{\text{M}}$	0.042	τ_{max}	1000
$\bar{g}_{\text{L}}$	0.12	$\bar{g}_{\text{T}}$	0
E_{leak}	-75	C_m	1
E_{Na}	50	E_{K}	-90
E_{Ca}	120	V_a	2
